# Complete loss of lower lip in a one-year-old treated with delayed free tissue transfer: a case report and comprehensive review of the literature

**DOI:** 10.1093/jscr/rjaf811

**Published:** 2025-10-14

**Authors:** Jacob Beiriger, Daniel E Bestourous, Nilam Patel, Reema Padia, Richard Cannon, Hilary C McCrary

**Affiliations:** Department of Otolaryngology–Head and Neck Surgery, University of Utah, 50 N. Medical Dr., SOM 3C120, Salt Lake City, UT 84132, United States; Department of Otolaryngology–Head and Neck Surgery, University of Utah, 50 N. Medical Dr., SOM 3C120, Salt Lake City, UT 84132, United States; Department of Otolaryngology–Head and Neck Surgery, University of Utah, 50 N. Medical Dr., SOM 3C120, Salt Lake City, UT 84132, United States; Department of Otolaryngology–Head and Neck Surgery, University of Utah, 50 N. Medical Dr., SOM 3C120, Salt Lake City, UT 84132, United States; Department of Otolaryngology–Head and Neck Surgery, University of Utah, 50 N. Medical Dr., SOM 3C120, Salt Lake City, UT 84132, United States; Department of Otolaryngology–Head and Neck Surgery, University of Utah, 50 N. Medical Dr., SOM 3C120, Salt Lake City, UT 84132, United States

**Keywords:** pediatric free flap reconstruction, lower lip avulsion, radial forearm free flap, static suspension, dog bite trauma, oral competence

## Abstract

Pediatric lower lip reconstruction is rare and technically challenging due to small donor sites, ongoing facial growth, and functional demands, such as speech and oral competence. We present the case of a one-year-old male who sustained complete avulsion of the lower lip from a dog bite. Initial management included a Karapandzic advancement flap for coverage. Definitive reconstruction at 23 months involved a radial forearm free flap with palmaris longus tendon for static suspension, augmented by acellular dermal matrix. Postoperative dehiscence required revision vestibuloplasty with a split-thickness skin graft. The patient achieved adequate lip suspension, improved sialorrhea, and restoration of oral competence by the first postoperative visit. Free flap reconstruction of the lower lip in children under two is feasible and can yield functional and aesthetic outcomes when individualized planning and multidisciplinary care are employed.

## Introduction

Lower lip reconstruction in young children poses significant challenges due to limited tissue, facial growth, and functional demands. Free flap use in pediatric patients remains rare, particularly in trauma-related facial defects [1, 2]. Flap selection must balance growth potential, donor site morbidity, and long-term outcomes [3, 4]. We present a case of total lower lip avulsion in a one-year-old child managed with staged reconstruction, including delayed free flap.

## Case

An 11-month-old male sustained a dog bite resulting in complete lower lip avulsion, mandibular exposure, and bilateral mental nerve injury. He underwent closure of facial lacerations, tracheotomy, gastrostomy tube placement, and dermal regeneration template placement to the lower face. He was transferred to our tertiary pediatric center for definitive care.

**Figure 1:**
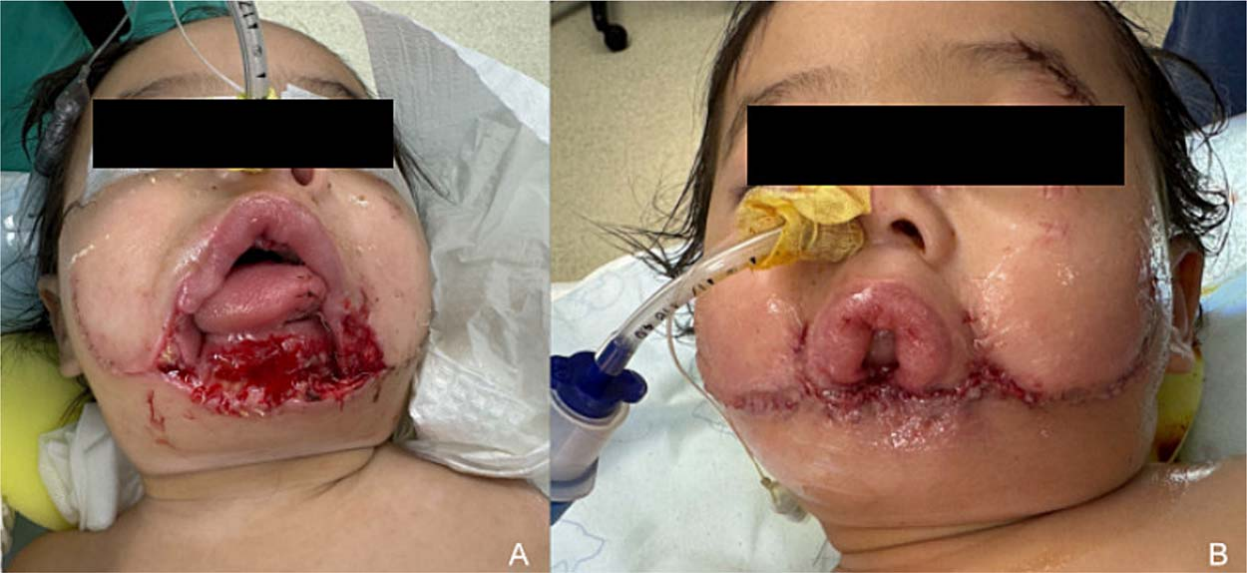
Initial presentation and local flap coverage. (a) Photograph upon initial presentation at 11 months of age to tertiary care facility demonstrating complete loss of lower lip with soft tissue defect exposing mandible. (b) Photograph following Karapandzic advancement flap coverage of mandible and resultant microstomia.

Operative evaluation revealed a 5 × 4 cm full-thickness lower lip defect, monocortical mandibular fracture, and intact oropharynx and larynx (Fig. 1a). A 10 × 6 cm Karapandzic advancement flap was performed for soft tissue coverage (Fig. 1b). A tracheotomy was placed due to persistent tongue edema and microstomia, and salivary botulinum toxin injection was performed to reduce sialorrhea. Definitive reconstruction was deferred to allow for growth.

**Figure 2:**
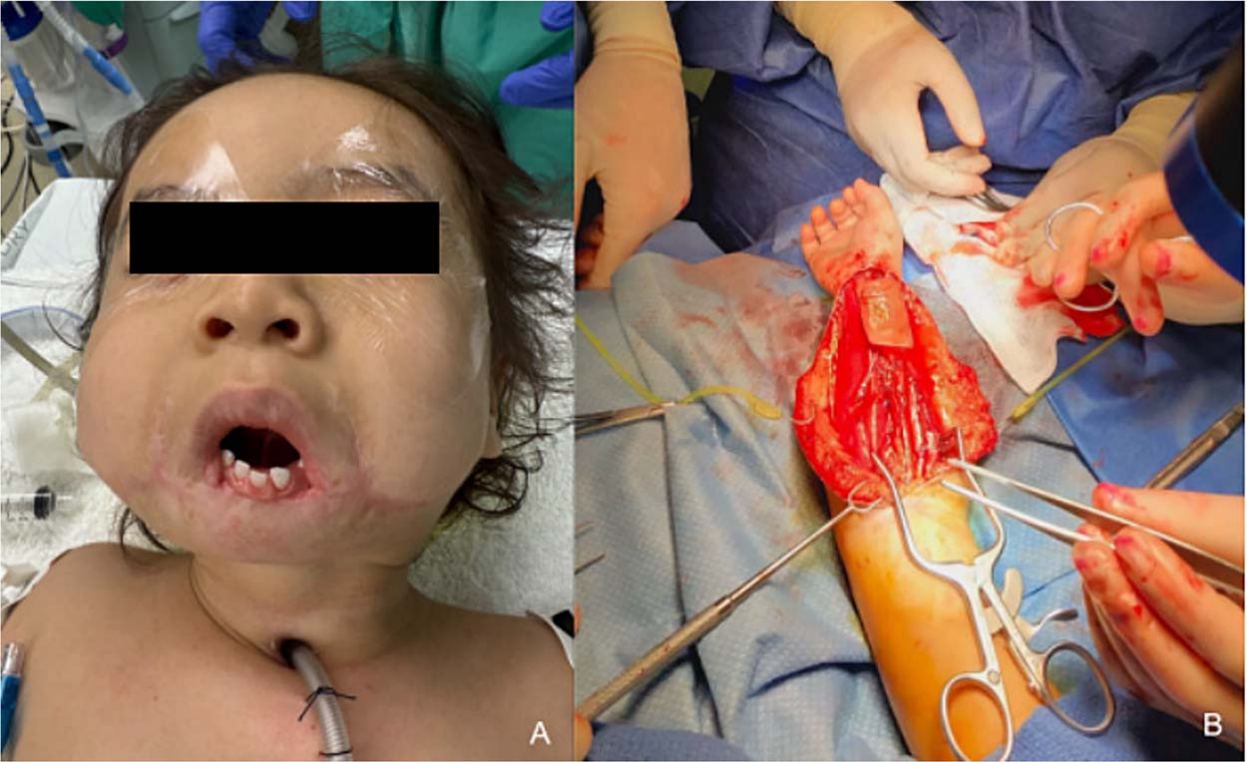
Preoperative evaluation and flap harvest. (a) Preoperative photograph (23 months of age) prior to definitive free flap reconstruction highlighting exposed mandibular dentition. (b) Intraoperative photo depicting harvest of the right RFFF.

**Figure 3:**
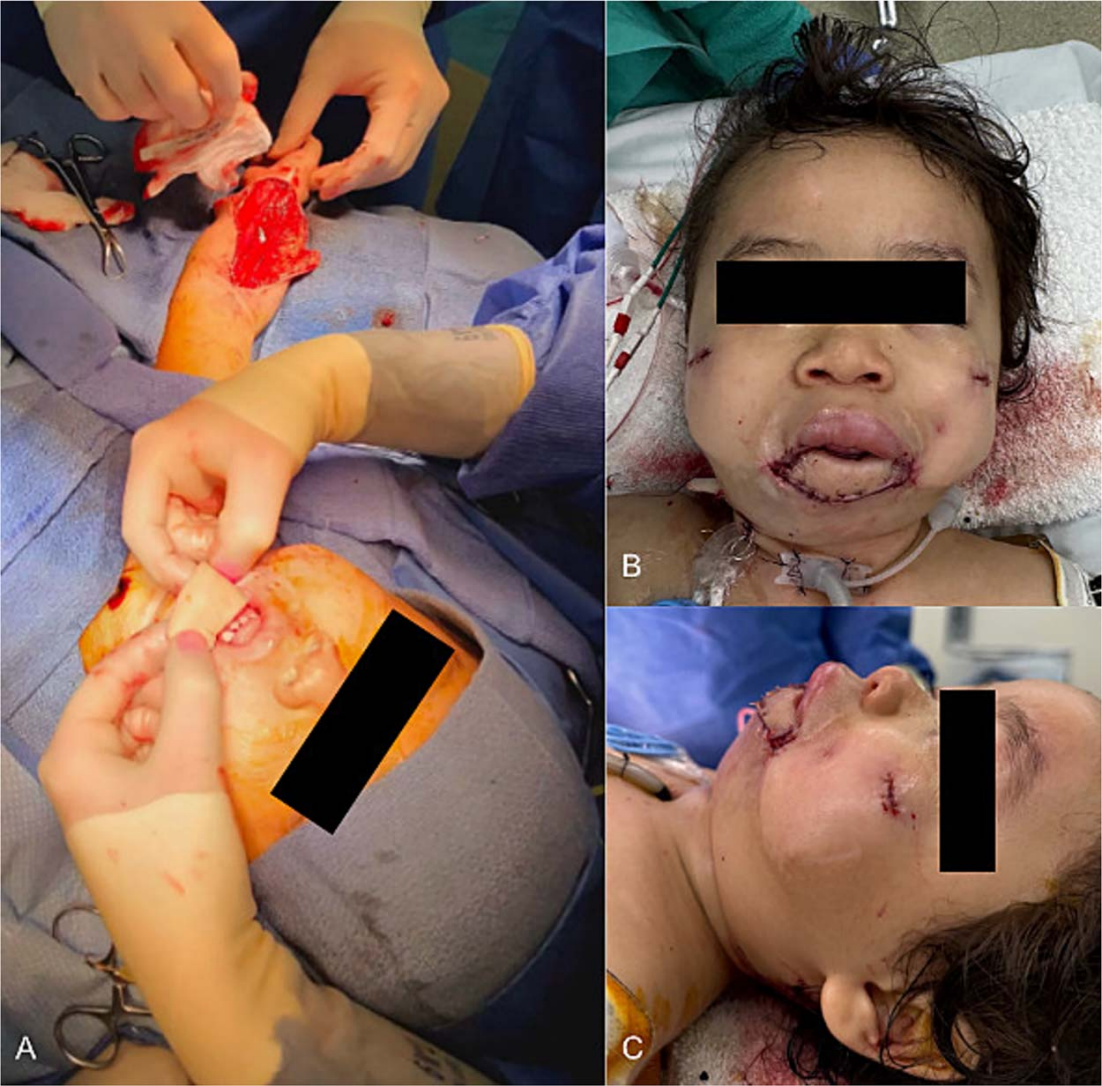
Inset of RFFF. (a) Intraoperative photo demonstrating the harvested RFFF positioned over the soft tissue defect. (b) Front profile following RFFF reconstruction of lower lip with suspension sutures to zygoma periosteum. (c) Side profile following RFFF reconstruction of lower lip.

At 23 months, the patient returned for reconstruction with a right radial forearm free flap (RFFF) and static sling using palmaris longus tendon and acellular dermal matrix (ADM) (Fig. 2a). A 6 × 4 cm musculocutaneous flap was harvested without tourniquet from the presumed non-dominant arm (Fig. 2b). The flap was inset externally and folded intraorally to recreate a gingivolabial sulcus and mucosal lip (Fig. 3a). The tendon was anchored to ADM and passed submucosally along the upper lip for suspension. Commissures were fixated to the zygomatic periosteum with absorbable suture (Fig. 3b and c). The pedicle was tunneled to the neck and anastomosed to the facial artery and external jugular vein. No perioperative complications occurred.

**Figure 4:**
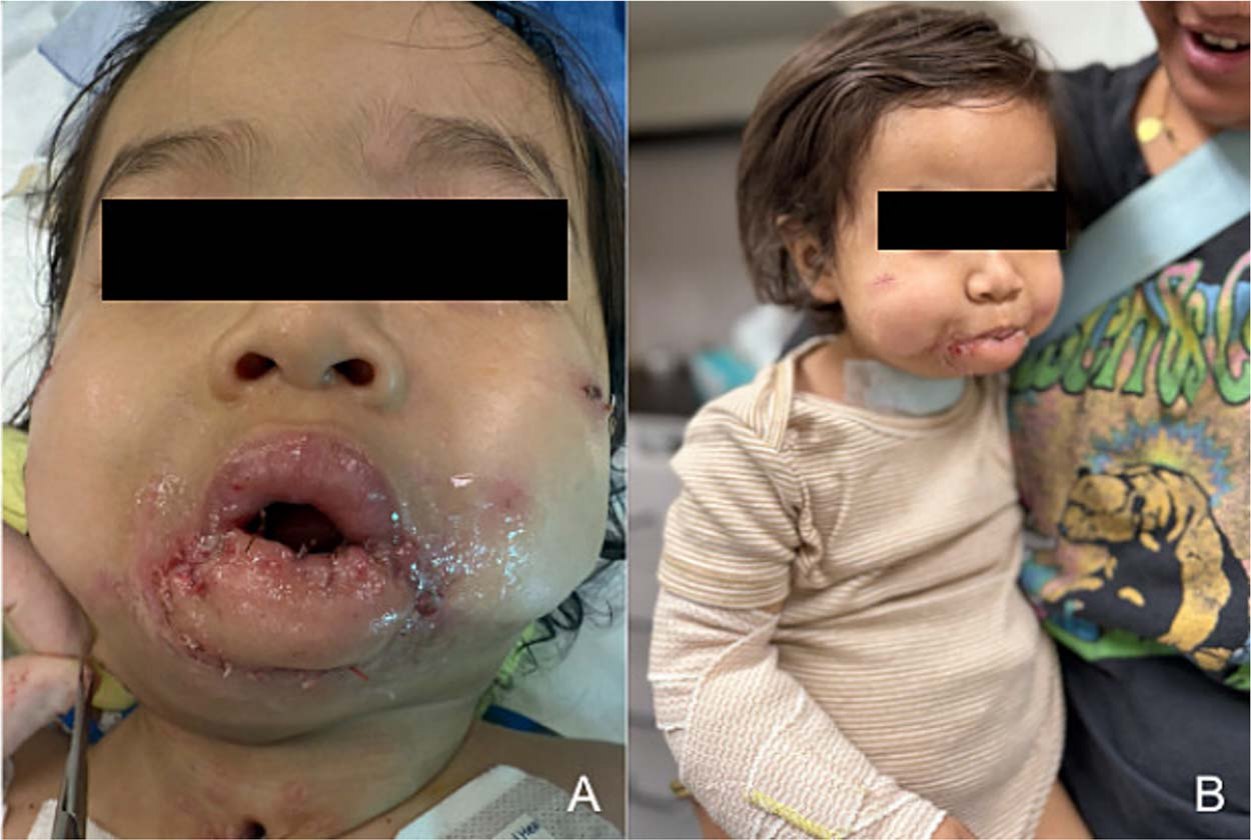
Postoperative outcome. (a) Postoperative photo demonstrating coverage of mandibular dentition following complex vestibuloplasty with split thickness skin graft and revision suspension of free flap to mandibular periosteum. (b) Patient photo from first postoperative check on POD 15 demonstrating mouth closure with lip opposition and restoration of oral competence.

On POD 6, the intraoral flap edge dehisced. On POD 7, the patient underwent revision vestibuloplasty using a split-thickness lateral thigh skin graft (Fig. 4a). Suspension sutures were placed to improve contour and coverage. He was decannulated and discharged without issue. At follow-up, he demonstrated full oral competence and improved sialorrhea (Fig. 4b).

## Discussion

Pediatric free flap reconstruction demands balance between functional restoration, aesthetics, and growth potential. The RFFF, anterolateral thigh (ALT) flap, and fibula flap are most frequently described in head and neck cases [3, 4, 5]. Flap selection is guided by patient age, defect complexity, and long-term goals.

Local options, such as Karapandzic, Abbe, or Estlander flaps, are effective for partial lip defects, preserving neurovascular integrity and allowing dynamic function. However, in infants with traumatic avulsion, local tissue is often insufficient for total lip reconstruction. Free flaps provide the necessary soft tissue bulk for large composite defects.

When dynamic oral competence is a priority, free functional muscle transfer (FFMT) using gracilis muscle has demonstrated success in both adults and children [6]. These techniques allow restoration of perioral animation when reinnervated with facial or masseteric nerve branches but require longer operative times, viable recipient nerves, and microsurgical expertise. In our patient, bilateral mental nerve injury and age precluded FFMT. Static suspension using tendon and ADM was chosen to minimize morbidity while restoring competence. Nonetheless, FFMT should be considered in older children with preserved innervation.

The RFFF is favored for lip reconstruction due to thin, pliable skin and tendon harvest capability [7, 8]. Its drawbacks include visible scarring, loss of a major hand vessel, and limited adaptability to growth [3]. Intraoperative Allen’s testing is required given difficulty performing it preoperatively in young children. Pediatric forearms pose size limitations, and the ulnar artery must be carefully protected, particularly when designing wider skin paddles. The ALT flap offers greater volume, favorable scarring, and low donor morbidity but lacks the contour for lip reconstruction. The fibula flap, while rarely used for lips, is valuable for mandibular and composite reconstruction, providing growth-compatible bone and minimal long-term donor complications [5].

Use of a tourniquet during flap harvest is debated. While it improves visualization, risks of ischemia and nerve injury may outweigh benefits in children. Decisions are typically individualized based on age and case complexity [3, 5]. Most pediatric free flaps are performed between ages 4–12, when donor sites are sufficiently developed [3, 4, 5]. However, successful harvests have been documented in infants, including a latissimus dorsi flap in a 6-month-old for temporal fossa reconstruction [9]. Early reconstruction is favored to promote optimal healing, prevent maladaptive habits, and improve psychosocial outcomes [5].

Delay in surgery should be dictated by clinical status—not donor site maturity. Both the ALT and fibula flaps demonstrate long-term adaptability with growth [3, 5]. Moreover, pediatric vessels tend to be free of atherosclerosis, less prone to vasospasm, and of favorable caliber for microanastomosis, offering technical advantages over adults [10].

Trauma-induced lip defects, especially from dog bites, require individualized evaluation. Early repair in children takes advantage of healing potential and minimizes the risk of persistent oral incompetence or social impact. Upton *et al.* [3] emphasizes avoiding prolonged delays, citing improved speech, swallowing, and facial growth with timely intervention. Starnes-Roubaud *et al.* [4] similarly reports superior functional and cosmetic results in early pediatric oncologic reconstruction. While some cases warrant delay for airway or infection control, waiting solely for child growth is not evidence-based.

In summary, reconstructive success in pediatric patients depends on matching defect characteristics with flap properties. The ALT flap offers bulk with minimal morbidity, the RFFF provides pliability and tendon support, and the fibula flap is ideal for bony components. Static suspension using tendon and ADM remains a valuable option when FFMT is not feasible. Our case demonstrates the importance of tailored, multidisciplinary planning in pediatric free flap reconstruction.

## Data Availability

All relevant data are included within the manuscript.
